# Accuracy of machine learning for differentiation between optic neuropathies and pseudopapilledema

**DOI:** 10.1186/s12886-019-1184-0

**Published:** 2019-08-09

**Authors:** Jin Mo Ahn, Sangsoo Kim, Kwang-Sung Ahn, Sung-Hoon Cho, Ungsoo S. Kim

**Affiliations:** 1 Department of Bioinformatics and Life Science, Soongsil University, Seoul, South Korea; 2 Functional Genome Institute, PDXen Biosystems Inc, Seoul, Republic of Korea; 3 Department of Ophthalmology, Kim’s Eye Hospital, Youngshin-ro 136, Youngdeungpo-gu, Seoul, 150-034 South Korea

**Keywords:** Machine learning, Pseudopapilledema, Optic neuropathy, Optic disc swelling

## Abstract

**Background:**

This study is to evaluate the accuracy of machine learning for differentiation between optic neuropathies, pseudopapilledema (PPE) and normals.

**Methods:**

Two hundred and ninety-five images of optic neuropathies, 295 images of PPE, and 779 control images were used. Pseudopapilledema was defined as follows: cases with elevated optic nerve head and blurred disc margin, with normal visual acuity (> 0.8 Snellen visual acuity), visual field, color vision, and pupillary reflex. The optic neuropathy group included cases of ischemic optic neuropathy (177), optic neuritis (48), diabetic optic neuropathy (17), papilledema (22), and retinal disorders (31). We compared four machine learning classifiers (our model, GoogleNet Inception v3, 19-layer Very Deep Convolution Network from Visual Geometry group (VGG), and 50-layer Deep Residual Learning (ResNet)). Accuracy and area under receiver operating characteristic curve (AUROC) were analyzed.

**Results:**

The accuracy of machine learning classifiers ranged from 95.89 to 98.63% (our model: 95.89%, Inception V3: 96.45%, ResNet: 98.63%, and VGG: 96.80%). A high AUROC score was noted in both ResNet and VGG (0.999).

**Conclusions:**

Machine learning techniques can be combined with fundus photography as an effective approach to distinguish between PPE and elevated optic disc associated with optic neuropathies.

## Background

Pseudopapilledema (PPE) is defined as an optic nerve with an elevated optic disc and blurred margins that is similar to papilledema or disc swelling associated with various optic neuropathies [1]. Although PPE is a benign condition, it should be differentiated from other optic neuropathies to reduce the need for unnecessary examination and to provide precise diagnosis, prognosis and therapeutic options to the patients. Recently, multi-modal imaging analysis including B-scan ultrasonography, fundus photography, autofluorescence, fluorescein angiography, and optical coherence tomography (OCT) have provided useful information for exact diagnosis of PPE [2–4]. However, the exact differentiation is still difficult.

Machine learning is the use of artificial computer intelligence to enable computers to learn automatically, without being programmed. In ophthalmology, machine learning has been used to analyze various disorders such diabetic retinopathy age-related macular degeneration, and glaucoma [5–8]. We investigated the accuracy and sensitivity of machine learning for differentiation between PPE, optic neuropathies and normals.

## Methods

### Patients

**Fig. 1:**
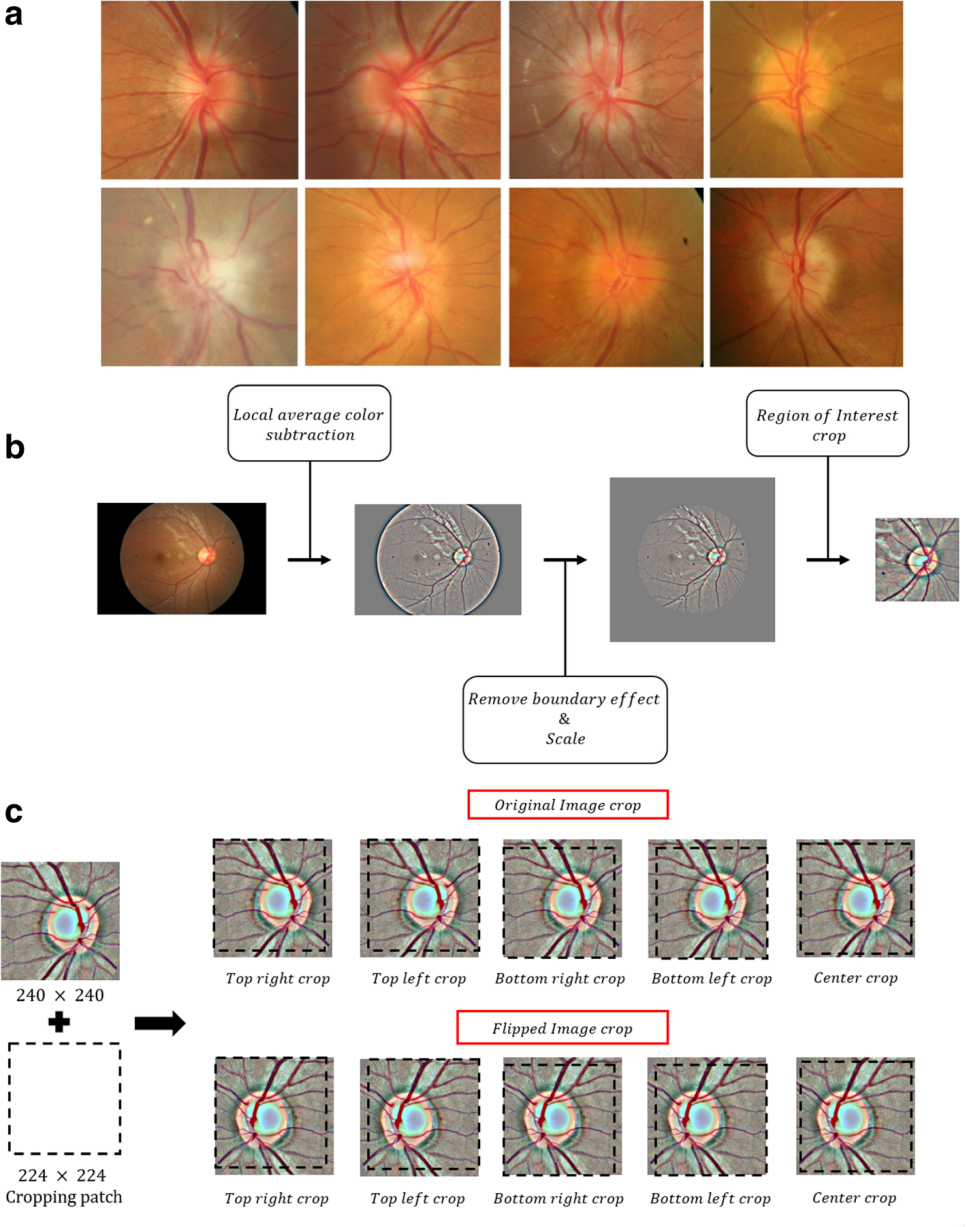
**a** Optic disc findings in fundus photography. Various features from pseudopapilledema (upper low) and swollen disc from optic neuropathies (lower low). **b** Schematic view of image pre-processing process **c** Schematic view of image augmentation process

Pseudopapilledema was defined as follows: cases with an elevated optic nerve head and blurred disc margins, with normal visual acuity (> 0.8 Snellen visual acuity), visual field, color vision, and pupillary reflex. Only those patients who did not change their optic nerve head and visual function for more than one year were included in the present study. The optic neuropathies group includes 177 cases of ischemic optic neuropathy, 48 of optic neuritis, 17 of diabetic optic neuropathy, 22 of papilledema, and 31 of retinal disorders such as central retinal vein occlusion or posterior uveitis (Fig. 1a). Normal controls were enrolled from routine examination without any abnormal findings and visual problems.

### Data preparation

Fundus photographs were collected from Kim’s Eye Hospital. Fundus photography was obtained using a non-mydriatic auto fundus camera (AFC-330, Nidek, Japan). A total of 1369 images were obtained, including 295 images of optic neuropathies, 295 of PPE, and 779 normal control images. The obtained images were scaled to a fixed width of 500 pixels while keeping the aspect ratio constant. To remove variations in lightning and brightness of images, the local average color was subtracted using Gaussian filtering [9]. Finally, pixels of each image was normalized to have 0 mean and 1 standard deviation. In order to produce fixed-size input necessary for machine learning models, each photos were cropped with size of 240 × 240 pixels at the region of optic nerve. Figure 1b shows the schematic view of the image pre-processing step. The entire set of 1369 images were split into an 876-image training dataset for training the model, a 274-image validation dataset for validation of the model while training, and a 219-image test dataset for evaluation of the final model. The validation dataset was generated by a random split of 20% of the entire dataset; the test dataset was generated by a random split of 20% of the remaining images after validation split (Table 1). Normal and PPE patients had normal findings on OCT (Cirrus HD-OCT, Carl Zeiss Meditec Inc., Dublin, CA and Spectralis, Heidelberg Engineering, Heidelberg, Germany), and visual field tests (Humphrey 740 visual field analyzer, Carl Zeiss Meditec Inc., Dublin, CA).

**Table 1:** Sample number for Convolutional Neural Network

	Normal	Pseudopapilledema	Papilledema	Total
Entire Data	779	295	295	1369
Training Data	505	197	174	876
Validation Data	155	53	66	274
Test Data	119	45	55	219

### Convolutional neural network

#### Data augmentation

To overcome possibility of overfitting the model due to small dataset, image augmentation technique was applied to each image. Augmentation process was conducted by cropping all four corners of an image and in the middle generating five images with a fixed size of 224 × 224 pixels. This cropping process was repeated after flipping the image which creates 10 augmented images from a single original image. Providing augmented images to a machine learning model can help overcome overfitting and to better in decision-making due to enlarged dataset with different pixel representation [10]. Figure 1c shows the shows augmentation process.

#### Training model

**Fig. 2:**
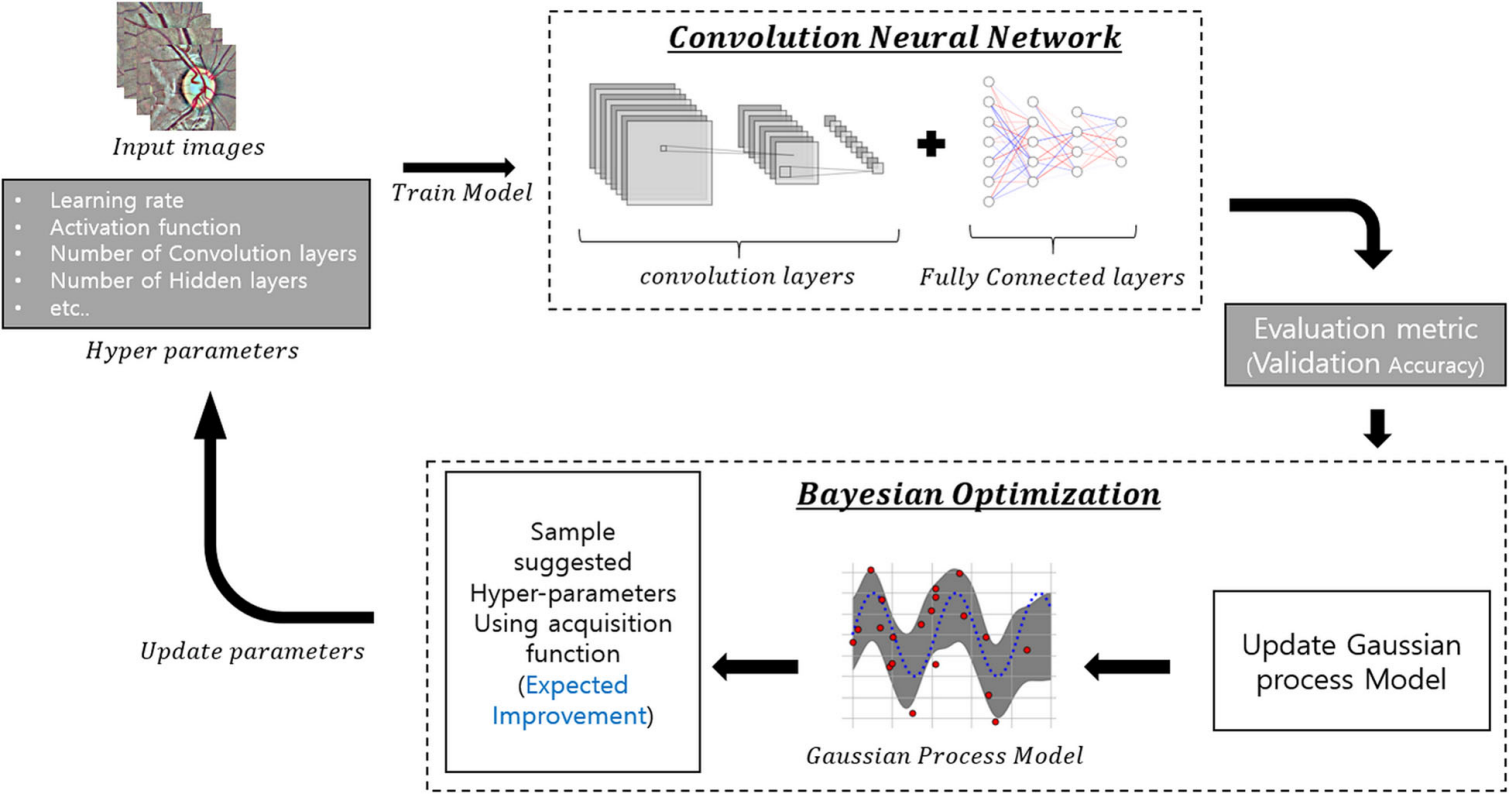
Schematic view of Bayesian optimization. Seven hyper-parameters were tuned using Bayesian optimization: learning rate, activation function, number of convolution layers, convolution patch size, filter size, number of fully connected layers, and number of hidden nodes in each fully connected layer

We have constructed a convolutional neural network, using Google’s Tensorflow deep learning framework as backend [11]. In order to produce best working model, an optimum set of working hyper-parameters are needed. These hyper-parameters include learning rate, activation function, patch size, filter size, number of fully connected layers, and number of hidden nodes in each fully connected layer. However, trying out all possible combinations of hyper-parameters is very time consuming and computationally expensive. Many methods have been proposed for hyper-parameter tuning such as grid search, random search [12], genetic algorithm [13], and Bayesian optimization [14]. We implemented Bayesian optimization for our hyper-parameter tuning process using python package Scikit-Optimize. Seven hyper-parameters were tuned using Bayesian optimization including number of convolution layers, number of convolution filters, number of convolution patch size, number of fully connected layers, number of hidden nodes in each fully connected layer, activation function (rectifier linear unit, exponential linear units, hyperbolic tangent), and learning rate. Max pooling layers were fixed after every convolutional layer with patch size 2 × 2 and stride 2, and dropout layers with rate 0.5 were fixed after every fully connected layer. Mattern kernel was used for Bayesian optimization and expected improvement was used for acquisition function. The best hyper-parameters were selected after 100 rounds of updating the Gaussian process model. Figure 2 shows a schematic view of hyper-parameter tuning process. The training was conducted again with the selected hyper-parameters with Adam optimizer [15] and cross entropy as a loss function until the average loss of validation data for each epoch started to increase.

#### Transfer learning

We conducted transfer learning [16], which involved training with a predefined (trained) model using three well-known convolutional neural networks. These include GoogleNet Inception v3 [17], 19-layer Very Deep Convolution Network from Visual Geometry group (VGG) and 50-layer Deep Residual Learning also known as ResNet [18, 19]. These networks were trained using approximately 1.2 million images from ImageNet Large-Scale Visual Recognition Challenge. We modified the fully connected layers of the three networks to fit our classification needs. Bayesian optimization was used to tune the hyper-parameters. Four hyper-parameters were tuned including number of fully connected layers, number of hidden nodes, activation function, and learning rate. Dropout layers with rate 0.5 were fixed after every fully connected layer. Fine-tuning was conducted after hyper-parameter tuning using Adam optimizer and cross entropy as a loss function. Training was considered finished when the average loss of validation data for each epoch started to increase.

### Evaluation

**Fig. 3:**
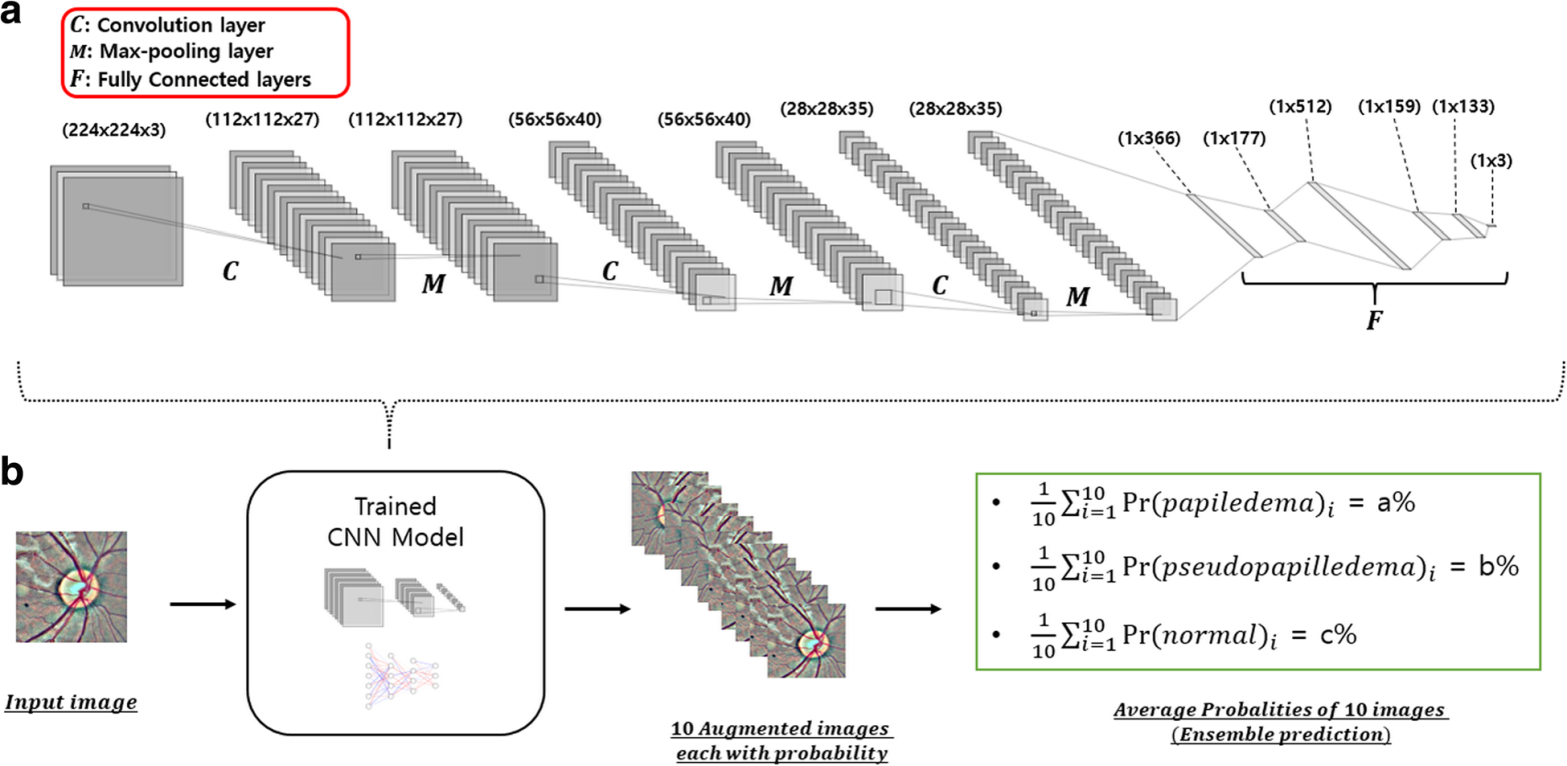
**a**. Schematic view of our model. It consists of 3 convolutional layer each with max pooling layer followed by 5 fully connected layers and a softmax layer. **b**. Evaluation process. Ten augmented images were averaged to give a single probability for each class

The model obtains an image and conduct mathematical calculation defined by training the model and ultimately outputs three probabilities. These probabilities represent a probability of being photograph of a normal subject, PPE and papilledema. Since we used augmented data (10 images per photography), we generated 30 probabilities (10 for each class) from a single image. By averaging these probability values by class, we obtained three probabilities which corresponds to image being normal, PPE and papilledema (Fig. 3b). The model makes decision by choosing the class with maximum probability. Using this strategy, we evaluated our model as well as GoogleNet Inception v3, VGG, and ResNet transferred model. Also, we have calculated micro-averaged sensitivity and specificity of each model and generated ROC (receiver operating characteristic) curve which indicates overall performance of how well the models classify images into three groups (Normal, PPE, papilledema).

## Results

Table 2 shows the summarized results of our model and transfer learning model. After hyper-parameter tuning, our model exhibited 3 convolution layers and 5 fully connected layers. The first convolution layer had a patch size of 2 × 2 with 27 filters, the second had a patch size of 12 × 12 with 40 filters, and the last convolution layer had a patch size of 29 × 29 with 35 filters. Max pooling layer was applied after every convolutional layer with a patch size of 2 × 2 and a stride of 2. The fully connected layers consisted of 366, 177, 512, 159, and 133 hidden nodes, respectively. A dropout rate of 0.5 was used in fully connected layers. As for activation function, ReLu (Rectifier linear unit) was used.

**Table 2:** Evaluation of our model and transferred model

	Our Model		Inception V3		ResNet		VGG	
	Ensemble Accuracy	AUROC	Ensemble Accuracy	AUROC	Ensemble Accuracy	AUROC	Ensemble Accuracy	AUROC
Training Data	100%	1.0	100%	1.0	100%	1.0	100%	1.0
Validation Data	96.35%	0.989	98.18%	0.993	98.18%	0.996	97.81%	0.996
Test Data	95.89%	0.992	96.35%	0.997	98.63%	0.999	96.80%	0.999

**Fig. 4:**
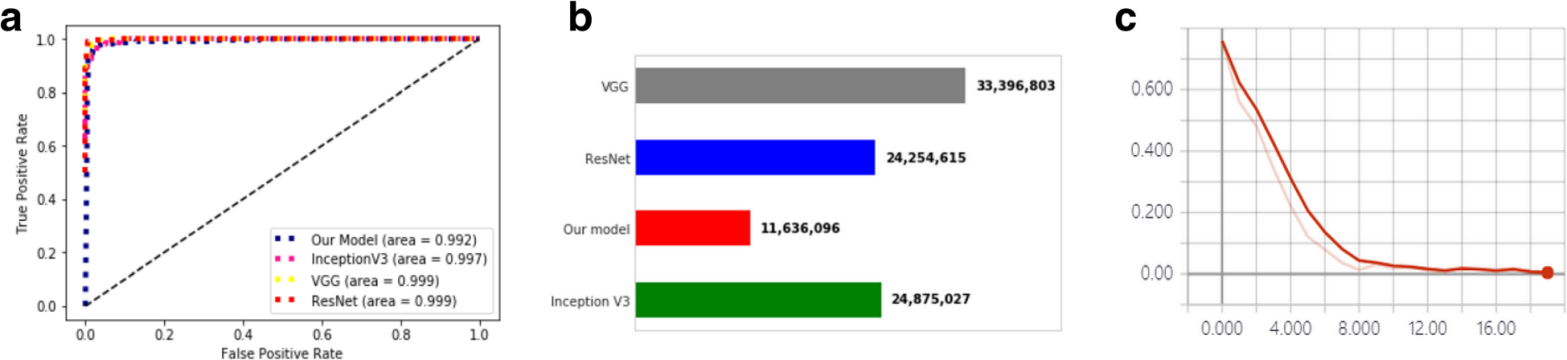
**a**. Receiver operating characteristic curve. **b**. Number of parameter comparison between models. **c**. Loss graph for our model. Y-axis indicates loss for validation data and X-axis indicates number of epoch

**Fig. 5:**
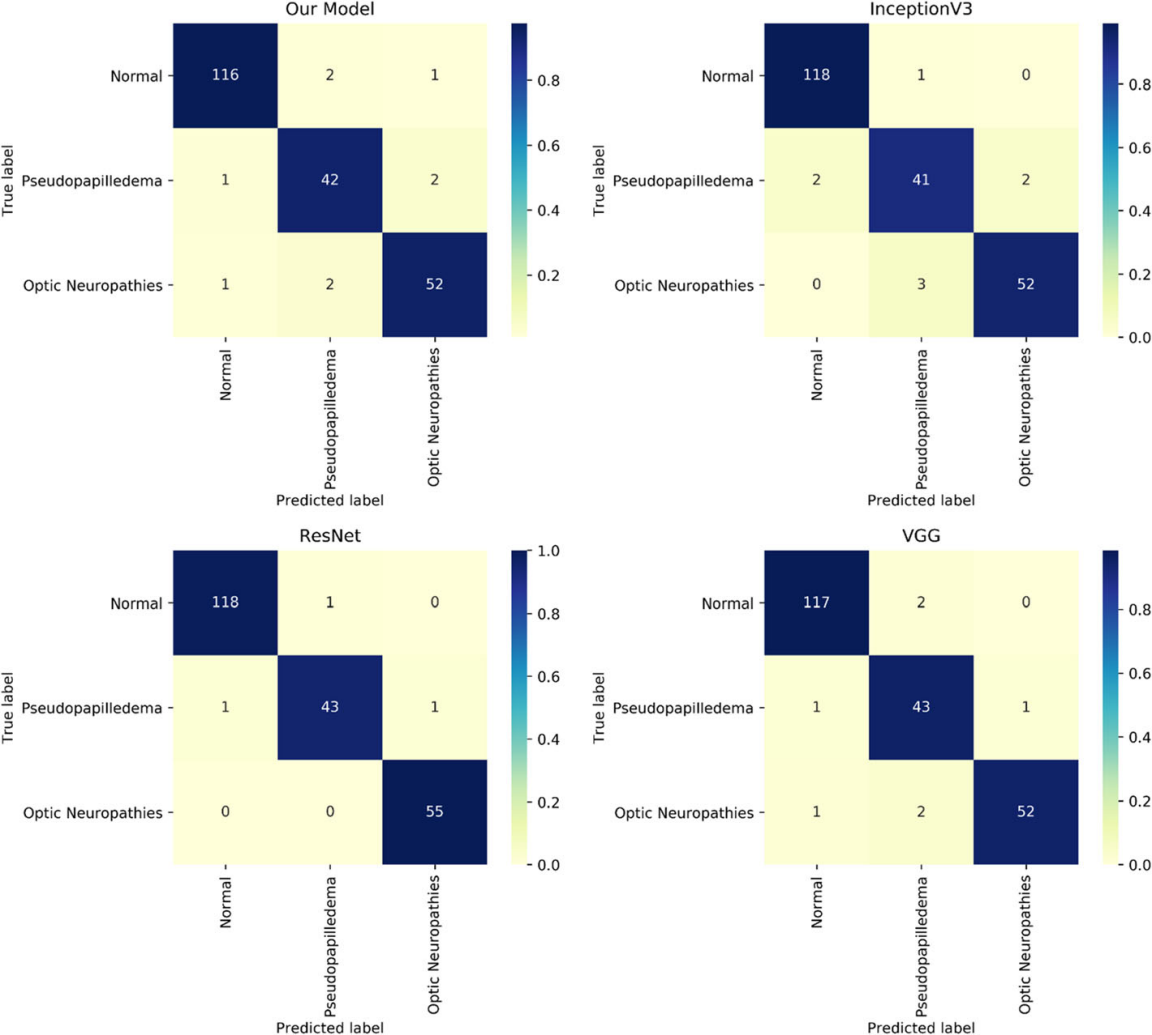
Confusion matrix

Figure 3a shows the schematic architecture of our CNN model. The Inception V3 model exhibited 1 fully connected layer with 60 hidden nodes along with Rectifier linear unit as an activation function. The VGG model exhibited 3 fully connected layers with each layer having 512 hidden nodes and Exponential linear unit as an activation function. The ResNet model had 1 fully connected layer with 325 hidden nodes with hyperbolic tangent as an activation function. All the transferred models had dropout layer with dropout rate 0.5 after every fully connected layer. Further, all models used softmax layer as a classification layer. The best performing model based on test accuracy was the ResNet transfer learned model. The ROC curve for each model is depicted in Fig. 4a and the confusion matrix based on test data for each model is depicted in Fig. 5. At the cost of 0.007 difference of AUROC, our model used the least number of parameters (11,636,096) among models (Fig. 4-b). In addition, the validation loss graph showed that validation loss reached zero level at around epoch 16 (Fig. 4c).

## Discussion

This study suggests that machine learning techniques can be combined with fundus photography as an effective approach to distinguish between PPE and elevated optic disc related with optic neuropathies.

We have used 3 state-of-the-art convolutional neural networks, including GoogleNet Inception V3, VGG, and ResNet. In addition, we used pre-trained weights from ImageNet Large-Scale Visual Recognition Challenge as the initial parameter to train our model instead of random weights; this is a popular method since these initial parameters are already optimized for detecting natural images such as edges and curves [20], thus solving the issue of overfitting when not many data are available. We have also trained our own model from scratch using Bayesian optimization as the hyper-parameter tuning process. As depicted in Table 2, transferred models outperformed our model based on test accuracy but, the difference, based on AUROC, was small. Between our model and the best performing ResNet transferred model, our model used far less parameters, which is computationally effective.

Overfitting, which refers to models performing well on the trained data but not well on unseen data is a common issue when a small dataset is used to train the model [21]. Since our dataset consisted of only 1369 images, there might have been a possibility of overfitting. However, we addressed this issue by incorporating regularization techniques such as adding dropout layers and data augmentation. Dropout randomly corrupts hidden nodes between layers which changes the detail of the model every training iteration [22]. Thus, this process leads to a more generalized model when a sufficient number of training iterations are given. Data augmentation allows the machine to learn an image from different views. This technique can also help overcome the issue of small training dataset by generating many augmented images. The validation loss graph for our model indicates that our model reached minimum, which is an indication of an optimal model [10].

Even though we have generated our own model and used well-known, state-of-the-art convolutional neural networks, getting an insight into how a machine classifies a fundus photo as normal or disease status can be a challenging task. Therefore, further study is needed into visualizing the convolution layers and filters to get an idea of how machines classify images. Further, larger scale datasets may help validate our findings.

## Conclusions

Machine learning techniques can be combined with fundus photography as an effective approach to distinguish between PPE and elevated optic disc associated with optic neuropathies.

## Data Availability

The datasets analyzed during the current study are available from the corresponding author (ungsookim@kimeye.com) on reasonable request.

## Abbreviations

OCT: Optical coherence tomography
PPE: Pseudopapilledema
VGG: Visual Geometry group
